# MyProstateScore in men considering repeat biopsy: validation of a simple testing approach

**DOI:** 10.1038/s41391-022-00633-3

**Published:** 2022-12-30

**Authors:** Jeffrey J. Tosoian, Michael S. Sessine, Bruce J. Trock, Ashley E. Ross, Cassie Xie, Yingye Zheng, Nathan L. Samora, Javed Siddiqui, Yashar Niknafs, Zoey Chopra, Scott Tomlins, Lakshmi P. Kunju, Ganesh S. Palapattu, Todd M. Morgan, John T. Wei, Simpa S. Salami, Arul M. Chinnaiyan

**Affiliations:** 1 Department of Urology, Vanderbilt University Medical Center, Nashville, TN USA; 2 Vanderbilt-Ingram Cancer Center, Nashville, TN USA; 3 Department of Urology, University of Michigan, Ann Arbor, MI USA; 4 Rogel Cancer Center, University of Michigan, Ann Arbor, MI USA; 5 Michigan Center for Translational Pathology, University of Michigan, Ann Arbor, MI USA; 6 Department of Urology, Wayne State University School of Medicine, Detroit, MI USA; 7 Department of Urology, Johns Hopkins University School of Medicine, Baltimore, MD USA; 8 Department of Urology, Northwestern Feinberg School of Medicine, Chicago, IL USA; 9 Fred Hutchinson Cancer Research Center, Seattle, WA USA; 10 Department of Pathology, University of Michigan, Ann Arbor, MI USA; 11 Howard Hughes Medical Institute, University of Michigan, Ann Arbor, MI USA

**Keywords:** Predictive markers, Cancer screening

## Abstract

**Background:**

Men with persistent risk of Grade Group (GG) ≥ 2 cancer after a negative biopsy present a unique clinical challenge. The validated MyProstateScore test is clinically-available for pre-biopsy risk stratification. In biopsy-naïve patients, we recently validated a straightforward testing approach to rule-out GG ≥ 2 cancer with 98% negative predictive value (NPV) and 97% sensitivity. In the current study, we established a practical MPS-based testing approach in men with a previous negative biopsy being considered for repeat biopsy.

**Methods:**

Patients provided post-digital rectal examination urine prior to repeat biopsy. MyProstateScore was calculated using the validated, locked model including urinary PCA3 and TMPRSS2:ERG scores with serum PSA. In a clinically-appropriate primary (i.e., training) cohort, we identified a lower (rule-out) threshold approximating 90% sensitivity and an upper (rule-in) threshold approximating 80% specificity for GG ≥ 2 cancer. These thresholds were applied to an external validation cohort, and performance measures and clinical outcomes associated with their use were calculated.

**Results:**

MyProstateScore thresholds of 15 and 40 met pre-defined performance criteria in the primary cohort (422 patients; median PSA 6.4, IQR 4.3–9.1). In the 268-patient validation cohort, 25 men (9.3%) had GG ≥ 2 cancer on repeat biopsy. The rule-out threshold of 15 provided 100% NPV and sensitivity for GG ≥ 2 cancer and would have prevented 23% of unnecessary biopsies. Use of MyProstateScore >40 to rule-in biopsy would have prevented 67% of biopsies while maintaining 95% NPV. In the validation cohort, the prevalence of GG ≥ 2 cancer was 0% for MyProstateScore 0–15, 6.5% for MyProstateScore 15–40, and 19% for MyProstateScore >40.

**Conclusions:**

In patients who previously underwent a negative prostate biopsy, the MyProstateScore values of 15 and 40 yielded clinically-actionable rule-in and rule-out risk groups. Using this straightforward testing approach, MyProstateScore can meaningfully inform patients and physicians weighing the need for repeat biopsy.

## Introduction

Diagnostic risk stratification is particularly challenging in patients who have previously undergone a negative prostate biopsy. The risk of detecting clinically-significant cancer (Grade Group [GG] ≥ 2) is reduced following a negative biopsy [1], and patients diagnosed on repeat biopsies have favorable risk profiles compared to those diagnosed on initial biopsy [1–3]. Moreover, the diagnostic value of PSA is limited in the repeat biopsy setting, given that the majority of patients undergo initial biopsy due to PSA elevation [4, 5]. Thus, the repeat biopsy setting is in particular need of new diagnostic tools to better inform the risk of harboring potentially-lethal cancer.

One option is the MyProstateScore (MPS) test (LynxDx, Inc., Ann Arbor, MI), which combines urinary expression of prostate cancer antigen 3 (PCA3) and the TMPRSS2:ERG gene fusion (T2:ERG) with serum PSA to define the risk of GG ≥ 2 cancer [6]. A growing body of data supports the association of MPS and clinico-pathologic endpoints, including cancer grade on biopsy [6, 7], grade and adverse pathology in radical prostatectomy specimens [8], and longer-term oncologic outcomes [9]. To facilitate clinical application of the MPS test, we recently validated a practical, threshold-based testing approach yielding 98% negative predictive value (NPV) and 97% sensitivity for GG ≥ 2 cancer in the biopsy-naïve setting [7]. While the MPS model has been shown to improve diagnostic accuracy relative to PSA-based risk calculators in both the initial and repeat biopsy settings [6], previous research has not outlined a clear, evidence-based testing approach in the repeat biopsy population [10].

Acknowledging the nuanced clinical challenge of men with persistent risk of GG ≥ 2 cancer following a negative biopsy, and the need for clear, data-driven guidance in clinical decision-making, we sought to validate a practical MPS-based testing approach for men considering repeat prostate biopsy.

## Materials/subjects and methods

### Threshold analysis

Considering the risk profile of the repeat biopsy population [1–3], we proposed a testing approach to identify both: (i) patients at low risk of GG ≥ 2 cancer that are very unlikely to benefit from repeat biopsy (rule-out testing), and (ii) patients at elevated risk of GG ≥ 2 cancer that are very likely to benefit from biopsy (rule-in testing). As previously described [7], considering the relative harms of false-positive and false-negative results [11] and the reduced risk profile of men with a previous negative biopsy [1–3], we sought a rule-out threshold approximating 90% sensitivity for GG ≥ 2 cancer. Acknowledging a more favorable risk profile in this setting and the desire to appropriately avoid biopsies not yielding GG ≥ 2 cancer, we sought an upper threshold conferring 80% specificity (i.e., avoiding 80% of unnecessary biopsies) [12, 13]. Additional details of this analysis are provided in the Supplementary Information.

### Study population and protocol

The MPS model was previously developed in 711 men presenting to three academic centers for prostate biopsy and validated in 1244 men across seven community clinics [6]. Study participants were referred for prostate biopsy based on elevated PSA and/or abnormal DRE, and those with prior treatment of PCa or prostate surgery were excluded. The primary (i.e., training) cohort for the current study included eligible men from these cohorts with a history of previous negative biopsy (*n* = 422). The external validation cohort of this analysis included 268 men scheduled for repeat prostate biopsy enrolled in an Early Detection Research Network study [14]. These cohorts have been previously described and are outlined in detail in the Supplementary Information [6, 7]. Notably, validation data were locked and accessible by only two study investigators (CX, YZ), who performed the analysis based on the pre-specified plan.

Institutional review board approval was obtained at participating sites, and all patients provided informed consent. First-catch post-DRE urine was prospectively-collected prior to biopsy, mixed with RNA stabilization buffer, and frozen to −70 °C per protocol [6]. *PCA3*, *T2:ERG*, and *PSA* mRNA were determined by transcription-mediated amplification, and PCA3 and T2:ERG scores were calculated by normalization to *PSA* mRNA, as previously described and detailed in the Supplementary Information [6]. MPS was calculated using established, locked-in models including only serum PSA, PCA3 score, and T2:ERG score. MPS values result on a continuous scale from 0 (very unlikely to detect GG ≥ 2 cancer) to 100 (very likely to detect GG ≥ 2 cancer). Patients underwent standard systematic prostate biopsy, defined as 10 or 12 cores without MRI guided targeting of high-risk lesions.

### Statistical analysis

The primary outcome was GG ≥ 2 cancer. Based on clinical rationale, we identified lower (rule-out) and upper (rule-in) thresholds in the primary cohort. Thresholds from the primary cohort were applied to the external validation population, and performance metrics (sensitivity, specificity, NPV, positive predictive value [PPV]) and potential clinical outcomes were calculated for each threshold. Clinical outcomes included: number (%) of biopsies avoided, number (%) of unnecessary (i.e., negative or GG1) biopsies avoided, and number (%) of GG ≥ 2 and GG ≥ 3 cancers missed (i.e., delayed) [11]. To supplement clinical rationale, we performed statistical analyses to ensure the clinically-derived thresholds were numerically reasonable (Supplementary Information) [7, 15]. Analyses were performed using Stata IC v16.1 and R version 3.6.

## Results

### Primary cohort and threshold identification

The primary study cohort included 422 men with a history of negative biopsy who underwent repeat biopsy. As listed in Table 1, the median age was 66 years (IQR 61–72), median PSA was 6.4 ng/ml (4.3–9.1), and median MPS was 23.1 (13.0–38.4). On biopsy, 58 men (14%) were found to have GG ≥ 2 cancer, and 25 men (5.9%) had GG ≥ 3 cancer. A lower threshold value of 15 best approximated the target sensitivity of 90%, and an upper threshold of 40 provided 80% specificity for GG ≥ 2 cancer. The prevalence of GG ≥ 2 cancer in the primary cohort was 5.1% for MPS ≤ 15, 13% for MPS values 15–40, and 27% for MPS > 40.

**Table 1:** Demographic and clinical characteristics of both primary and validation cohorts.

	Primary Cohort	Validation Cohort
	Median (IQR) or *N* (%)	Median (IQR) or *N* (%)
*N*	422	268
Age (years)	66 (61–72)	64 (59–69)
African American race	27 (6.4%)	26 (9.7%)
Positive family history	85 (21%)	67 (25%)
Suspicious DRE	61 (15%)	32 (12%)
PSA (ng/ml)	6.4 (4.3–9.1)	7.6 (5.5–10.6)
MPS	23.1 (13.0–38.4)	28.9 (16.9–49.2)
Biopsy results		
Negative	282 (67%)	205 (76%)
GG1	82 (19%)	38 (14.2%)
GG ≥ 2	58 (14%)	25 (9.3%)
Biopsy results		
Negative	282 (67%)	205 (76%)
GG1	82 (19%)	38 (14.2%)
GG2	33 (7.8%)	11 (4.1%)
GG3	9 (2.1%)	5 (1.9%)
GG4	8 (1.9%)	6 (2.2%)
GG5	8 (1.9%)	3 (1.1%)

### External validation of threshold values

The validation cohort included 268 men of median age 64 years (59–69), median PSA 7.6 ng/ml (5.5–10.6), and median MPS 28.9 (16.9–49.2) (Table 1). Most men (57%) underwent repeat biopsy for elevated PSA, and a complete summary of indications is provided in the Supplementary Information. Repeat biopsy was negative in 205 men (76%), revealed GG1 cancer in 38 men (14%), and demonstrated GG ≥ 2 cancer in 25 men (9.3%). Furthermore, 14 men (5.2%) had GG ≥ 3 disease. Among men with MPS values 0–15 in the validation cohort, no patients (0%) were found to have GG ≥ 2 cancer on biopsy. In the 124 men with MPS values 15–40, eight (6.5%) had GG ≥ 2 cancer, and 4 (3.2%) had GG ≥ 3 disease. In the remaining 89 patients with MPS values >40, 17 men (19%) had GG ≥ 2 disease and 10 (11.2%) had GG ≥ 3. The prevalence of GG ≥ 2 cancer in the primary and validation cohorts is listed by MPS category in Table 2.

**Table 2:** Prevalence of GG ≥ 2 prostate cancer on repeat biopsy by MPS categories in primary and validation cohorts.

	*Primary Cohort*		*Validation Cohort*	
MPS	*N* (%)	GG ≥ 2 PCa	*N* (%)	GG ≥ 2 PCa
≤15	137 (33%)	7 (5.1%)	55 (21%)	0 (0%)
15–40	186 (44%)	24 (13%)	124 (46%)	8 (6.5%)
>40	99 (23%)	27 (27%)	89 (33%)	17 (19%)
Total	422	58 (14%)	268	25 (9.3%)

### Validated performance of MPS thresholds and potential clinical outcomes

In the external validation population, MPS values ≤15 ruled out GG ≥ 2 cancer with 100% NPV and 100% sensitivity. Applying a MPS threshold of 15 to rule-out biopsy would have avoided 21% of biopsies, while missing zero GG ≥ 2 cancers (0%). Using the upper MPS threshold of 40 to rule-in biopsies for only men at highest risk (i.e., MPS > 40) would have avoided 179 biopsies (67%) and maintained a 95% negative predictive value, while delaying the diagnosis of eight GG ≥ 2 cancers (32%). For GG ≥ 3 cancer, the upper threshold of 40 provided a negative predictive value of 98% and sensitivity of 71%. The performance measures and clinical outcomes associated with the use of MPS thresholds 15 and 40 are listed in Table 3.

**Table 3:** Performance and clinical outcomes of MPS testing for GG ≥ 2 prostate cancer for MPS thresholds of MPS ≤ 15 and MPS > 40 in the validation cohort.

	Sn	Sp	NPV	PPV	*N* (%) bx avoided	*N* (%) unnecessary bx avoided	*N* (%) GG ≥ 2 diagnoses missed	*N* (%) GG ≥ 3 diagnoses missed
Validation cohort (*n* = 268)								
MPS ≤ 15	100%	23%	100%	12%	55 (21%)	55 (23%)	0 (0%)	0 (0%)
MPS > 40	68%	70%	95%	19%	179 (67%)	171 (70%)	8 (32%)	4 (29%)

*Sn* sensitivity, *Sp* specificity, *NPV* negative predictive value, *PPV* positive predictive value, *bx* biopsy.

## Discussion

Men with a previous negative biopsy and a persistent risk of clinically-significant cancer offer a unique clinical challenge. While multiple tools have been proposed for risk stratification, the vast majority of validation data are derived from the biopsy-naïve setting [10]. In the current analysis, we established and validated a straightforward clinical approach to MPS-based risk stratification in the repeat biopsy population. In an independent validation cohort, we found that MPS ≤ 15 was associated with 100% NPV and 100% sensitivity for GG ≥ 2 cancer. By contrast, men with MPS > 40 had an approximately one in five risk of GG ≥ 2 disease. The intermediate range of MPS 15–40 was associated with a 6.5% risk of GG ≥ 2 disease. In the setting of shared decision-making, these data suggest that men with MPS ≤ 15 can confidently forego biopsy (0% risk of GG ≥ 2 PCa), most men with MPS 15–40 are unlikely to benefit from repeat biopsy (6.5% risk of GG ≥ 2 PCa), and those with MPS > 40 harbor risk of GG ≥ 2 cancer (19%) that supports proceeding to repeat biopsy [16].

There are limited data describing the use of biomarkers in clearly-defined repeat biopsy populations [10]. McKiernan and colleagues assessed various thresholds of the ExoDx Prostate (*Intelliscore*), or EPI test, in one cohort of 229 men with PSA 2-10 ng/ml that underwent repeat biopsy. Consistent with our study population and the available literature, 12% of their cohort had GG ≥ 2 cancer on repeat biopsy. Using the EPI score of 15.6, they found that 27% of unnecessary repeat biopsies could have been avoided with 82% sensitivity and 92% NPV for GG ≥ 2 disease [17]. As validated herein, the lower MPS threshold of 15 would have prevented 23% of unnecessary biopsies with 100% sensitivity and 100% NPV for GG ≥ 2 disease. An upper EPI threshold of 29.6 would have avoided 65% of unnecessary biopsies with 68% sensitivity and 94% NPV. Similarly, we found that the upper MPS threshold of 40 would have avoided 70% of unnecessary biopsies, with 68% sensitivity and 95% NPV. Moreover, the upper MPS threshold of 40 provided 98% NPV for GG ≥ 3 disease. As such, available data suggest that biomarker assays are likely to provide clinically useful information to the majority of men considering repeat biopsy.

Multiparametric MRI (mpMRI) is similarly proposed to better inform patients considering repeat biopsy. Unlike biomarkers, a positive mpMRI can allow clinicians to target visible lesions, improving the yield of biopsy [18]. By contrast, mpMRI is subject to several practical limitations. For one, MRI is not an option for some patients (e.g., metallic implants, claustrophobia) and is not universally accessible, emphasizing the need for multiple diagnostic testing options. Most notably, MRI is subjectively interpreted, and its accuracy varies across and within medical centers [19, 20]. A 2020 meta-analysis found that there were insufficient data to calculate the diagnostic accuracy of MRI in the non-academic setting [19]—where the majority of patients receive care. Among academic centers, the NPV for PI-RADS 1–2 mpMRI ranged from 67% to 100%, with a pooled NPV of 90.8%, and the proportion of patients that would have avoided biopsy based on negative MRI ranged by study from 3% to 67%. Acknowledging the limitations of cross-study comparisons and that the cited MRI data are not specific to the repeat biopsy setting, it is notable that the MPS threshold of 40 would have avoided 67% of biopsies—the highest proportion observed across MRI studies—with 95% NPV. Thus, MPS appears to offer potential as an objective, expandable, cost-effective tool that can be routinely obtained in the urology clinic [21].

There are notable limitations of the current analysis. For one, we were unable to assess test performance by specific indication for repeat biopsy due to the limited number of events. Second, patients in the current analysis underwent standard systematic prostate biopsy, which results in underdetection of GG ≥ 2 cancer relative to the pathologic gold standard (i.e., prostatectomy specimen). Importantly, however, recent data support that MPS is strongly associated with surgical pathology findings in men with low-risk biopsy features [8], further corroborating its association with clinically-meaningful endpoints, including long-term oncologic outcomes [9]. It is also notable that the current analysis was performed outside the context of mpMRI. Yet these findings support use of MPS as a standalone test to rule out the need for additional testing prior to MRI or biopsy. As such, the absence of MRI is appropriate for the proposed clinical application. Furthermore, despite the increasing role of MRI in diagnostic evaluation of prostate cancer, pre-biopsy MRI is still only performed in a minority of patients in the United States (16.7% from 2017 to 2019) [22]. Thus, characterizing the performance of biomarkers as standalone tests—to potentially avoid MRI or biopsy—is critical to better understanding their role in clinical practice. While the validation cohort was limited in size and events, the number of participants exceeds similar published repeat biopsy cohorts, and the event rate is consistent with published repeat biopsy populations [17]. Ultimately, this study population reflects one of the largest repeat biopsy cohorts to undergo biomarker testing under a prospective, standardized biospecimen protocol.

In conclusion, the current study provides a straightforward, validated clinical application of the MPS test in patients with a previous negative biopsy considering repeat biopsy. In a clinically-appropriate validation population, the MPS threshold of 15 was 100% sensitive for GG ≥ 2 cancer, providing a highly reliable test to safely rule out the need for repeat biopsy. At the same time, MPS values >40 were associated with an approximately one in five risk of GG ≥ 2 disease—effectively identifying those higher-risk patients that stand to benefit most from repeat biopsy. Further studies will aim to corroborate these findings and define practical testing approaches across additional clinical settings.

## Supplementary information

Supplement

Supplementary Figure 1

## Data Availability

JJT and AMC had full access to all primary cohort data and take full responsibility for the integrity of the data and the accuracy of the data analysis. These data are available from the corresponding author on reasonable request. CX and YZ had full access to all validation cohort data and take full responsibility for the integrity of the data and the accuracy of the data analysis. These data are available upon reasonable request and with permission of the NCI Early Detection Research Network.
