# Supplementary material for: MyProstateScore in men considering repeat biopsy: validation of a simple testing approach
Source: Prostate Cancer Prostatic Dis. 2022 Dec 30;26(3):563–7. doi: 10.1038/s41391-022-00633-3 (PMC10310885; doi:10.1038/s41391-022-00633-3)
Supplement: Supplementary file 1 — Supplement [file 41391_2022_633_MOESM1_ESM.docx]

**MyProstateScore in Men Considering Repeat Biopsy: Validation of a Simple Testing Approach**

**SUPPLEMENT**

Tosoian, Sessine, Trock, et al.

**Study Population**

The primary cohort of the current analysis was derived from prospective cohorts participating in initial MPS training and validation. The initial training cohort included 711 men participating at one of three academic medical centers (﻿Dana Farber Harvard Cancer Center; Cornell University; and University of Michigan) [1]. Median (interquartile range, IQR) age was 63 (57-68), median PSA was 5.1 ng/ml (3.8-7.0), 99 men (14%) had a suspicious digital rectal examination (DRE), and 174 men (24%) had a history of previous negative biopsy. On biopsy, 192 men (27%) had GG≥2 cancer. The validation cohort of the initial MPS study included 1225 men referred for biopsy at seven US community clinics [1]. Median age was 64 (58-70), median PSA was 4.7 (3.3-6.5), 287 (23%) had a suspicious DRE, and 248 (20%) had undergone a previous negative biopsy. On biopsy, 224 men (18%) had GG≥2 cancer. As such, the primary cohort of the current analysis included the 422 participants with a history of a previous negative biopsy. Demographic and clinical characteristics of this primary cohort are provided in the primary report.

The validation cohort of the current analysis was derived from a population of 859 men prospectively-enrolled at 11 academic centers as part of an Early Detection Research Network validation trial of PCA3 [2]. Mean (SD) age was 62 years (8), PSA was 8 ng/ml (14), and 141 men (16%) had an abnormal DRE. On biopsy, GG≥2 cancer was detected in 174 men (20%). Of the 297 patients with a previous negative biopsy, 268 men (90%) had conclusive T2:ERG results and were thus eligible for MPS assessment. Demographic and clinical characteristics of the final validation cohort of this analysis are provided in the primary report.

**MyProstateScore (MPS) Testing**

Urinary *T2:ERG, PCA3,* and *PSA* mRNA were quantified by transcription-mediated amplification (TMA) assays at the University of Michigan, Gen-Probe, Inc., and the ﻿EDRN Biomarker Reference Laboratory at Johns Hopkins. As previously described [1], T2:ERG scores were determined by normalization of *T2:ERG* mRNA expression to *PSA* mRNA expression using the following formula: (100 000 × average urine *TMPRSS2:ERG* copies/mL) / (average urine *PSA* copies/mL). Samples with mean urinary *PSA* >10 000 copies/mL were considered informative. Similarly, the Progensa PCA3 assay was used to quantify *PCA3* and *PSA* mRNA expression. PCA3 score is calculated as 1 000 × (average urine *PCA3* copies/mL) / (average urine *PSA* copies/mL). Samples with mean urine *PSA* >7 000 copies/mL were considered informative. All laboratories were blinded to prostate biopsy results. MPS values were calculated based on the previously published, locked model including T2:ERG score, PCA3 score, and serum PSA:

*logit(p)=-5.8588+0.59038*LOG(1+PSA,2)+0.55316*LOG(1+PCA3,2)+0.14371*LOG(1+T2:ERG,2)*

**Statistical Calculations**

As described, clinically-relevant MPS thresholds were identified in the primary cohort and further evaluated in the validation cohort. Prostate biopsy results were used to classify MPS values as true positive (TP), true negative (TN), false positive (FP), and false negative (FN) relative to the proposed thresholds. Performance measures were determined by standard calculations for sensitivity (TP / TP+FN), specificity (TN / TN+FP), negative predictive value (TN / TN+FN), and positive predictive value (TP / TP+FP) [3, 4]. The proportion of biopsies avoided was calculated as the number of negative tests divided by the number of patients. The proportion of unnecessary biopsies avoided (i.e. specificity) was calculated as the number of true negative tests divided by the total number of patients without GG≥2 cancer. The proportion of GG≥2 cancers missed or delayed was calculated as the number of patients with negative tests found to have GG≥2 cancer divided by the total number of GG≥2 cancers.

**Defining Risk Categories: Threshold Analysis**

Previous authors have described why statistical methods alone are inappropriate for identifying cutpoints [5]. Specifically, numerical methods do not account for the clinical consequences of incorrect categorization (i.e. false positives, false negatives). Therefore, consistent with our previous analysis in biopsy-naïve patients [6], the current approach to categorization was based primarily on clinical rationale and subsequently confirmed as numerically reasonable using the statistical approaches of Williams et al. [7].

*Clinical Rationale*

As such, we sought a threshold to rule-out GG≥2 cancer with sufficiently high sensitivity to preclude the need for biopsy in the majority of patients with a negative test. Previously published risk models have proposed sensitivity of 90-95% for GG≥2 cancer as reasonable in this clinical setting [8], and our previous validation of MPS in biopsy-naïve patients identified a threshold value conferring 95% sensitivity [6]. Given the more favorable risk profile of patients diagnosed with cancer following a prior negative biopsy [9], the current analysis sought a threshold value providing 90% sensitivity in patients undergoing repeat biopsy. At the same time, given the reduced likelihood of aggressive PCa in the repeat biopsy population, we sought an additional threshold to minimize false positive testing and reduce unnecessary biopsies, while “ruling in” those patients at highest risk of harboring GG≥2 PCa that are most likely to benefit from repeat biopsy. To that end, we proposed a threshold conferring 80% specificity [10].

*Numerical Assessment*

While risk categorization was primarily based on clinical reasoning, previous authors have provided statistical approaches to assess the numerical appropriateness of cutpoints. Williams and colleagues at the Mayo Clinic Department of Health Sciences Research offer that the appropriateness of cutpoint models are reasonably supported when a monotonic relationship is observed between the continuous covariate and the binary outcome [6]. Minimally, a dichotomy of outcome probability should be observed between low and high values of the covariate. **Supplementary** **Figure 1** plots the proportion of patients experiencing the outcome (GG≥2 cancer) across incremental increases in MPS values in the primary cohort (n=422), ensuring reasonable sample sizes (n≥15) of subgroups. The monotonic relationship of MPS and GG≥2 cancer is apparent. Similarly, grouped data support that the proposed risk strata are numerically reasonable [7]. **Supplementary Table 1** lists the prevalence of GG≥2 cancer across MPS values. All MPS sub-categories 0-15 were associated with a prevalence of GG≥2 cancer ≤6.3%. Consistent with this, a maximum regression-based probability of GG≥2 cancer of 8.1% was observed at the MPS value of 15. By contrast, the prevalence of GG≥2 cancer exceeded 20% for all MPS sub-categories above 40, and the predicted probability of GG≥2 cancer exceeded 20% for all MPS values greater than 45. Acknowledging the potential for categorization to over-simplify the underlying model, these data suggest that the proposed risk categories reasonably reflect the continuous risk model and are consistent with the clinical rationale described.

**Supplementary Table 1.** Prevalence of GG≥2 cancer by increasing MPS values in the primary cohort.

| **MPS** | **N** | **GG≥2** |
| --- | --- | --- |
| **≤5** | 18 | 0 (0%) |
| **5-10** | 55 | 3 (5.5%) |
| **10-15** | 64 | 4 (6.3%) |
| **15-20** | 50 | 5 (10%) |
| **20-25** | 40 | 4 (10%) |
| **25-30** | 40 | 9 (18%) |
| **30-35** | 28 | 3 (11%) |
| **35-40** | 19 | 3 (16%) |
| **40-45** | 17 | 4 (24%) |
| **45-50** | 17 | 4 (24%) |
| **>50** | 65 | 19 (29%) |

**Supplementary Table 2.** Performance and clinical outcomes of MPS testing for GG≥2 prostate cancer for MPS thresholds of MPS ≤15 and MPS >40 in the primary cohort.

|  | **Sn** | **Sp** | **NPV** | **PPV** | **N (%) bx avoided** | **N (%) unnecessary bx avoided** | **N (%) GG≥2 diagnoses missed** | **N (%) GG 3+** |
| --- | --- | --- | --- | --- | --- | --- | --- | --- |
| Primary cohort (n=422) | | | | | | | |  |
| MPS ≤15 | 88% | 36% | 95% | 18% | 137 (32%) | 152 (36%) | 7 (12%) | 2 (8.1%) |
| MPS >40 | 47% | 80% | 90% | 27% | 323 (77%) | 338 (80%) | 31 (53%) | 12 (48%) |

**Supplementary Table 3.** Indications for repeat biopsy in the validation cohort.

| **Indication for biopsy** | **N** |
| --- | --- |
| Abnormal DRE | 30 (11%) |
| PSA >2.0 ng/ml | 152 (57%) |
| Elevated PSA velocity (>0.4 ng/ml/yr) | 57 (21%) |
| Lower PSA value with other risk factors for PCa (e.g. family history) | 1 (0.4%) |
| Prior ASAP or HGPIN | 19 (7.1%) |
| % free PSA <15% | 2 (0.7%) |
| Other | 7 (2.6%) |
| Total | 268 |

Additional Information

**Supplementary Figure and Table Captions and Legends**

Supplementary Table 1. Prevalence of GG≥2 cancer by increasing MPS values in the primary cohort.

Supplementary Table 2. Performance and clinical outcomes of MPS testing for GG≥2 prostate cancer for MPS thresholds of MPS ≤15 and MPS >40 in the primary cohort.

Supplementary Table 3. Indications for repeat biopsy in the validation cohort.

Supplementary Figure 1. Observed prevalence of GG≥2 cancer on biopsy by MPS Values in the primary cohort.

Supplementary Figure 1 Legend: Percentage of patients with GG≥2 prostate cancer (Y-axis) within respective MPS ranges (X-axis). Vertical lines represent discussed threshold values of 15 and 40.

REFERENCES

1. Tomlins SA, Day JR, Lonigro RJ, Hovelson DH, Siddiqui J, Kunju LP, et al. Urine TMPRSS2:ERG Plus PCA3 for Individualized Prostate Cancer Risk Assessment. Eur Urol. 2016;70(1):45-53.

2. Wei JT, Feng Z, Partin AW, Brown E, Thompson I, Sokoll L, et al. Can urinary PCA3 supplement PSA in the early detection of prostate cancer? J Clin Oncol. 2014;32(36):4066-72.

3. Florkowski CM. Sensitivity, specificity, receiver-operating characteristic (ROC) curves and likelihood ratios: communicating the performance of diagnostic tests. Clin Biochem Rev. 2008;29 Suppl 1:S83-7.

4. Tosoian JJ, Ross AE, Sokoll LJ, Partin AW, Pavlovich CP. Urinary Biomarkers for Prostate Cancer. Urol Clin North Am. 2016;43(1):17-38.

5. Assel M, Sjoberg D, Elders A, Wang X, Huo D, Botchway A, et al. Guidelines for Reporting of Statistics for Clinical Research in Urology. Eur Urol. 2019;75(3):358-67.

6. Tosoian JJ, Trock BJ, Morgan TM, Salami SS, Tomlins SA, Spratt DE, et al. Use of the MyProstateScore Test to Rule Out Clinically Significant Cancer: Validation of a Straightforward Clinical Testing Approach. J Urol. 2021;205(3):732-9.

7. Williams BA, Mandrekar JN, Mandrekar SJ, Cha SS, Furth AF. Finding Optimal Cutpoints for Continuous Covariates with Binary and Time-to-Event Outcomes. Technical Report Series. 2006; 79.

8. Aladwani M, Lophatananon A, Ollier W, Muir K. Prediction models for prostate cancer to be used in the primary care setting: a systematic review. BMJ Open. 2020;10(7):e034661.

9. ElShafei A, Nyame Y, Kara O, Badawy A, Amujiogu I, Fareed K, et al. More Favorable Pathological Outcomes in Men with Low Risk Prostate Cancer Diagnosed on Repeat versus Initial Transrectal Ultrasound Guided Prostate Biopsy. J Urol. 2016;195(6):1767-72.

10. Ferraro S, Bussetti M, Bassani N, Rossi RS, Incarbone GP, Bianchi F, et al. Definition of Outcome-Based Prostate-Specific Antigen (PSA) Thresholds for Advanced Prostate Cancer Risk Prediction. Cancers (Basel). 2021;13(14).
