# Supplementary figures and images for: MyProstateScore in men considering repeat biopsy: validation of a simple testing approach
Source: Prostate Cancer Prostatic Dis. 2022 Dec 30;26(3):563–7. doi: 10.1038/s41391-022-00633-3 (PMC10310885; doi:10.1038/s41391-022-00633-3)

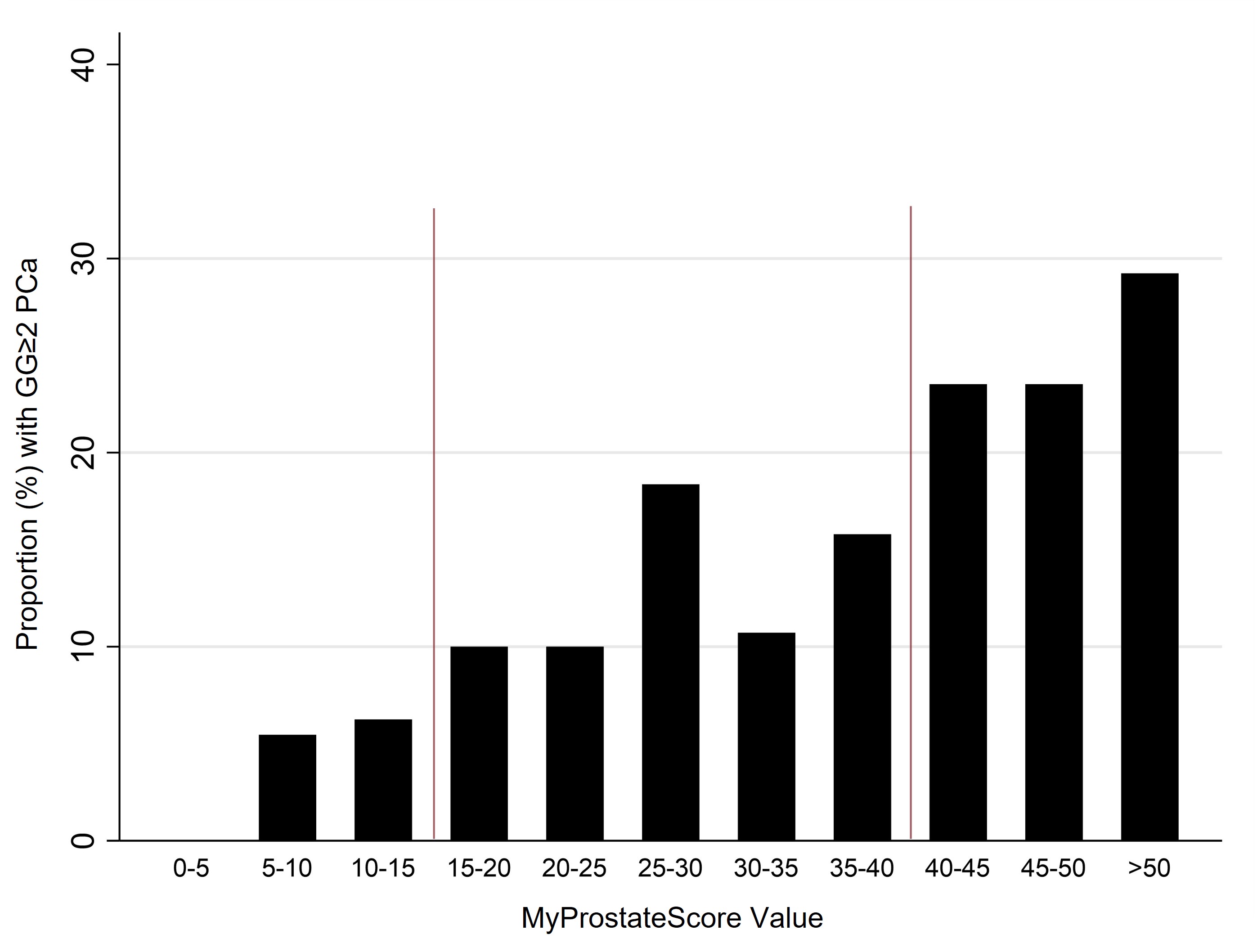

Supplement: Supplementary file 2 — Supplementary Figure 1 [file 41391_2022_633_MOESM2_ESM.jpg]
